# To Stick or Not to Stick: Adhesions in Orofacial Clefts

**DOI:** 10.3390/biology11020153

**Published:** 2022-01-18

**Authors:** Angelo Antiguas, Brian J. Paul, Martine Dunnwald

**Affiliations:** Department of Anatomy and Cell Biology, The University of Iowa, Iowa City, IA 52245, USA; angelo-antiguas@uiowa.edu (A.A.); brian-paul@uiowa.edu (B.J.P.)

**Keywords:** palatogenesis, cleft lip and palate, morphogenesis, development, cytoskeleton, adhesions, epithelial cells, craniofacial development

## Abstract

**Simple Summary:**

During embryonic development, cells proliferate and move to form structures essential to each organ. As these dynamic processes occur cells must form adhesions with their neighbors. These adhesions serve as communication centers to amplify the behavior of individual cells into collective cellular events. Here, we review cell-to-cell adhesions and focus on their role in the development of the face and palate.

**Abstract:**

Morphogenesis requires a tight coordination between mechanical forces and biochemical signals to inform individual cellular behavior. For these developmental processes to happen correctly the organism requires precise spatial and temporal coordination of the adhesion, migration, growth, differentiation, and apoptosis of cells originating from the three key embryonic layers, namely the ectoderm, mesoderm, and endoderm. The cytoskeleton and its remodeling are essential to organize and amplify many of the signaling pathways required for proper morphogenesis. In particular, the interaction of the cell junctions with the cytoskeleton functions to amplify the behavior of individual cells into collective events that are critical for development. In this review we summarize the key morphogenic events that occur during the formation of the face and the palate, as well as the protein complexes required for cell-to-cell adhesions. We then integrate the current knowledge into a comprehensive review of how mutations in cell-to-cell adhesion genes lead to abnormal craniofacial development, with a particular focus on cleft lip with or without cleft palate.

## 1. Introduction

Morphogenesis is a complex process in which cells divide, move, and interact with one another and extracellular matrices. These events are highly coordinated across a range of timescales and physical spaces, resulting in the formation of tissues and organs that are essential to a functional body. Morphogenesis is highly dynamic; it integrates mechanical forces with biochemical signals, both sensed and produced by cells, to inform individual cellular behavior. At the cross-roads of force signaling and cell fate is the internal cytoskeleton and its remodeling, which, through the intermediary of cell junctions, amplify individual cell behavior into collective movement, which is critical for the development of organisms.

Amongst all developmental processes, the morphogenesis of the face is one of the most fascinating. It transforms the cranial part of the neural tube by way of distinct embryological processes that form the eyes, nose, chin, and mouth, each defining the identity of the organism. Not only is this morphogenic event extremely dynamic, but it also has the unique characteristic that most of the events are visible from the outside, allowing for a direct appreciation of the cellular movements and interactions. When the coordinated interplay of cellular migration, adhesion, and proliferation is disrupted it can lead to structural birth defects, such as orofacial clefts.

In this review we focus on craniofacial morphogenesis, with a particular emphasis on the formation of the lip and palate, and examine how the structures interacting with the cytoskeleton—namely cell–cell junctions—contribute to the morphogenesis of these tissues in humans and mice. We discuss the need to study the cytoskeletal rearrangements that regulate biomechanical processes to understand how cells move and adhere during embryonic development.

## 2. Craniofacial Morphogenesis

The development of the cranium and face is a highly complex process. It involves the precise spatial and temporal coordination of the migration, adhesion, growth, differentiation, and apoptosis of cells originating from the ectoderm, mesoderm, and endoderm. The result of these morphogenic events is a constellation of structures that is distinctly recognizable as human and critical to our interaction with the world.

### 2.1. Early Embryogenesis

**Figure 1:**
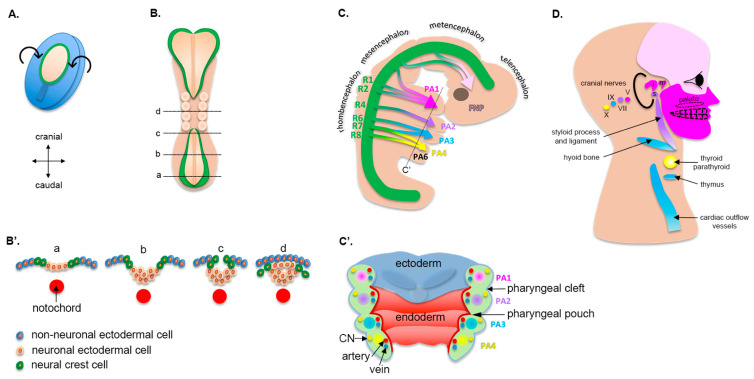
**Morphogenesis of pharyngeal arches and their derivatives.** (**A**) Dorsal view of a human embryo at approximately 16 d EGA. The neural plate is being specified (pale area) and will undergo dorsal folding. (**B**) Dorsal view of a human embryo at about 22 d EGA. Note that the neural folds (green color) start to fuse in the midline. Transverse views at different levels of the embryo are shown in (**B′**). (**C**) Lateral view of a human embryo at approximately 32 d EGA. The neural tube (green) is specified in different rhombomeres (R1 to R8), all of which (except R3 and R5) will contribute to pharyngeal arches. (**C′**) Longitudinal view of the pharyngeal arches. Each pharyngeal arch is composed of a core mesenchyme derived from the neural crest, which includes a unique cranial nerve, an artery, and a vein (see Table 1 for details). The narrowing between two pharyngeal arches is called a cleft (on the outside of the embryo) or a pouch (on the inside of the embryo). (**D**) Derivatives of pharyngeal arches in a human adult. CN = cranial nerve; i = incus; m = maleus; and s = stapes.

One of the critical early events of embryogenesis is the transition from a bilaminar to a trilaminar embryo. The addition of the third embryological layer starts at an estimated gestational age (EGA) of 14 days, with the formation of the primitive streak [1]. This streak is the result of the differentiation of ectodermal cells into rapidly dividing mesenchymal cells, starting at the caudal end of the embryo and moving cranially. These cells proliferate and migrate to fill the space between the endoderm and the ectoderm, with the exception of the oropharyngeal and cloacal membranes. Further differentiation of the primitive streak leads to the formation of the notochord, which serves as the embryonic axial skeleton and as a signaling center to the overlying ectoderm to differentiate into the neural plate (Figure 1A,B,B′).

The specification of the neural plate from the ectoderm occurs at 17 d EGA. At 22 d EGA the borders of the neural plate, also called neural folds, converge at the dorsal midline and begin to fuse, forming the neural tube (Figure 1B). First, the fusion occurs in the cranial region between the 3rd and 5th somite, which corresponds to what will eventually become the occipital region. Then, the fusion of the remaining neural folds proceeds caudally to complete the formation of the neural tube along the body axis (Figure 1B,B′). The failure of fusion in either direction results in the pathologies broadly termed as neural tube defects (for example, anencephaly from failure in the cranial direction and spina bifida caudally), some of the most common birth defects. In the context of this review, it is notable that there is an increased incidence of neural tube defects in individuals with orofacial clefts, indicating an overlap in risk factors for these two structural birth defects [2].

### 2.2. Cranial Neural Crest Cells

Cells that reside at the dorsal edge of the neural tube (i.e., crest of the neural tube), where the neural folds fused, are called neural crest cells (NCCs, Figure 1B,B′) [3]. These cells are specified at the border of the neural plate and the non-neural ectoderm via signaling gradients, which include WNT, BMP, and FGF, along with a large gene regulatory network of transcription factors. Following induction, NCCs undergo an epithelial-to-mesenchymal transition (EMT). They lose their cell-to-cell contact by decreasing E-cadherin levels, activate mechanosensitive molecules, including the small Rho GTPases and YAP, and migrate following the stimulation of growth factors such as WNT, BMP, and FGF [4]. NCCs ultimately differentiate into a variety of connective, muscular, nervous, endocrine, and pigmentary tissue, and induce the differentiation of overlying tissues that they invade. The reader is invited to read reviews on NCCs for extensive details [3,5].

Neural crest cells are grouped based on their anatomical position along the body axis, i.e., cranial, cardiac, or trunk. In the context of craniofacial development, cranial neural crest cells (CNCCs) are the key determinant of facial morphogenesis (Figure 1C). The migration of CNCCs from the cranial neural tube to ventral swellings and the first pharyngeal arch is considered the initial event of craniofacial development [4]. These cells determine the structures that will develop in each arch (Figure 1C,C′). Eventually, they form the facial cartilage, bone, dentin, muscles and tendons, and dermis, along with the forebrain, midbrain, hindbrain, and sensory neurons (Figure 1D, Table 1). Consequently, the embryological origin of all these structures in the face (CNCCs) is different than that of the rest of the body (often the mesoderm).

### 2.3. Pharyngeal Arches

The pharyngeal arches (or branchial arches, from the Greek *branchia* for *gill*) are bilateral pairs of swellings (or outpouchings) of the mesoderm that surround the developing pharynx of the embryo. They project forward from the dorsal aspect of the embryo and fuse in the ventral central midline (Figure 1C). Pharyngeal arches develop in a cranial-to-caudal sequence, with the first pharyngeal arch being the most cranial and initiating its morphogenesis around 20 d EGA, followed by the second and the third. By the time that the last arches develop the first two are no longer visible externally, as they progressed through morphogenesis. Consequently, diagrams, including ours, which show distinct arches at the same stage of development are, in fact, not an accurate representation of morphogenesis.

The first arch, as the first to form, separates the mouth pit (or stomodeum) from the externally developing pericardium. By differential growth, the neck elongates and new arches form, such that the embryo ultimately has six arches (Figure 1C). The first four are the most prominent and clinically relevant. The fifth pharyngeal arch involutes quickly after it is formed and is not relevant for subsequent development. The central core of each pharyngeal arch is composed of mesoderm-derived tissue, in which CNCCs migrate as they delaminate from the neural crest (Figure 1C′). As such, this tissue has a mixed embryological origin and is often referred to as the ecto-mesenchyme. Surrounding the ecto-mesenchyme are the endoderm-derived (medially) and ectoderm-derived (laterally) epithelia. At the junction of each arch the ectoderm and endoderm layers come closer to each other, but remain always separated by an ecto-mesenchymal layer. They form recesses called pouches on the endodermal side in addition to grooves or clefts on the lateral ectodermal surface (Figure 1C′) [6,7].

Each arch develops its own neurovascular structures that supply a distinct muscle group and skeletal tissue [8]. Derivatives of each arch (its core and associated pouch and cleft) are unique and listed in detail in Table 1. The first two arches are the most relevant for this review, as they give rise to the major bony structures of the middle ear, upper neck, and face, including the mandible (via Meckel’s cartilage), the maxilla, and the muscles of mastication and facial expression that overlie the bony structures. The last three pairs of arches give rise to the bones, muscles, and glands of the neck as well as the outflow tract of the heart, including the aortic arch and pulmonary arteries.

### 2.4. Formation of the Upper Lip, Nose, and Primary Palate

**Figure 2:**
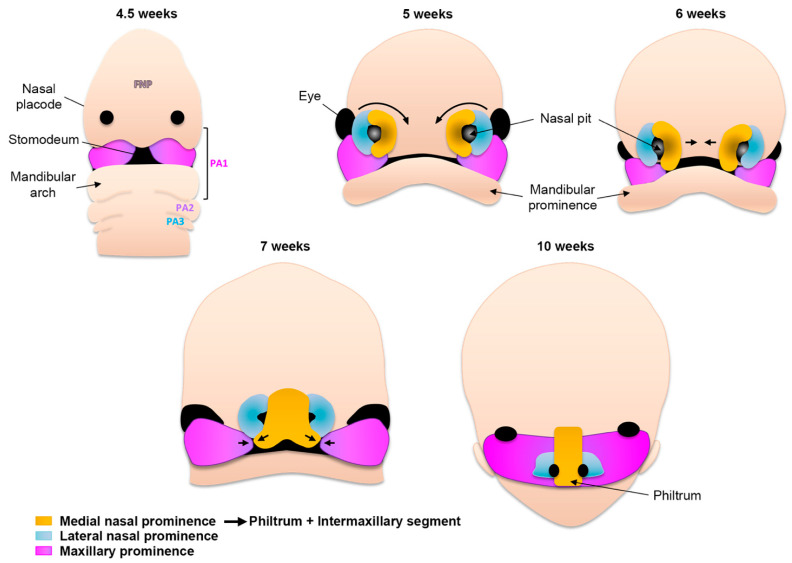
**Schematic of human facial morphogenesis (frontal views).** At 4.5 weeks the frontonasal prominence (FNP) constitutes the most rostral part of the embryo. Derivatives of the first pharyngeal arch (PA) contribute structures below the nasal placodes, including the mandibular arch. Note the presence of the stomodeum (primitive mouth). At 5 weeks the thickening of nasal placodes gives rise to medial and lateral prominences. Medial rotation (6 weeks) and fusion (7 weeks) of the medial prominences give rise to the philtrum of the lip and the intermaxillary segment (10 weeks). The lateral prominences contribute to the nasal ala.

At the end of the fourth week of gestation, as embryogenesis progresses, the unpaired frontonasal prominence becomes distinct on the ventral side of the forebrain. In addition, the first and second pharyngeal arches continue to swell ventrally and give rise to two pairs of processes: the maxillary and mandibular, both surrounding the primitive mouth (stomodeum) (Figure 2). Collectively, it is the well-orchestrated proliferation and migration of CNCCs in these five prominences as well as the specific tissue fusions between them that will lead to the formation of the face [7]. On both sides of the frontonasal prominence, local thickening of the ectoderm leads to the formation of nasal placodes (Figure 2). During the fifth week of gestation, these nasal placodes invaginate and become depressed nasal pits. At the same time, mesenchymal cells proliferate around the placodes, and the sides of these swellings form the medial and lateral nasal prominences. As the maxillary prominences continue growing they merge laterally with the mandibular prominences to form the cheeks. Medially, the growth of the maxillary prominences compresses the medial nasal prominences and causes them to fuse around the 10th week of development (Figure 2). Hence, the upper lip is formed by the two medial nasal prominences and the two maxillary prominences. This fusion creates the intermaxillary segment, composed of a labial component that forms the philtrum of the upper lip, an upper jaw component with the four incisors, and a palatal component that forms the triangular primary palate. The lateral nasal prominences give rise to the nasal alae, and their interaction with the maxillary prominences forms the nasolacrimal groove and nasolacrimal duct system.

### 2.5. Formation of the Secondary Palate

**Figure 3:**
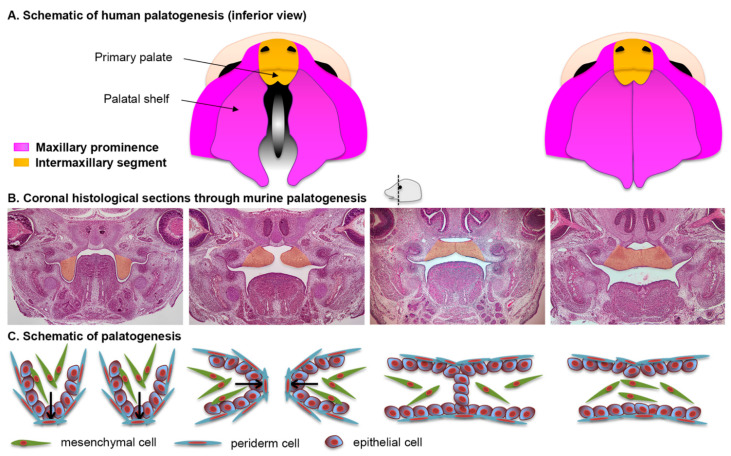
**Palatal morphogenesis**. (**A**) Schematic of human palatogenesis from an inferior view. (**B**) Coronal sections of murine embryonic heads stained with hematoxylin and eosin. The palatal shelves, highlighted in orange, change from a vertical position (approximately E13.5) to a horizontal position (approximately E14); their epithelial cells adhere to eventually leave a confluent bridge of mesenchymal cells (approximately E15). (**C**) Schematic representation of the histological views in (**B**).

**Figure 4:**
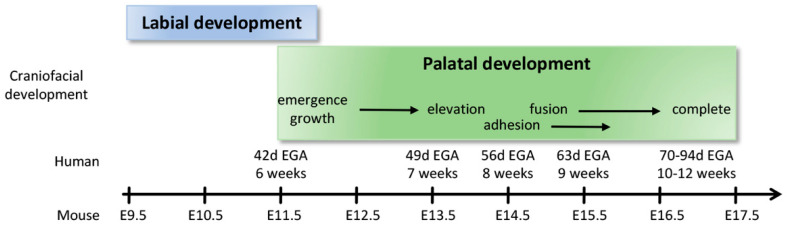
Timeline correlation between human and murine labial and palatal development.

The secondary palate is formed by pair-wise outgrowths from the maxillary prominences (Figure 3A). The reader is invited to consult complementary reviews related to human [9] and murine [10] palatogenesis. At around the sixth week of gestation in humans (E11.5 in mice, see Figure 4 for timeline correlation between mice and humans) these outgrowths, namely the palatine shelves, appear on each side of the tongue and begin to grow downward (Figure 3B,C). In the seventh week (E13.5–E14 in mice) these shelves reorient themselves and acquire a horizontal position as the tongue depresses inferiorly. At this stage the palatine shelf is composed of an ecto-mesenchyme (CNNC-derived) core surrounded by an epithelium (ectodermal-derived) with at least two layers of oral keratinocytes: a basal layer and a superficial squamous layer called the periderm (Figure 3C, see below for details). As the two palatine shelves come into closer proximity the palatal epithelia make contact in the midline. For adhesion, rather than just contact, to occur the periderm must slough off from the oppositional surfaces, and epithelial cells from each of the shelves intercalate to form a single-layered epithelial seam (termed medial edge epithelium or medial edge seam, MEE or MES) (Figure 3B,C) [11]. From a transverse view, this initial adhesion occurs in the middle of the secondary palate and proceeds anteriorly and posteriorly, similar to a “zipper” closing in both directions, ending at the incisive foramen anteriorly and the uvula posteriorly (Figure 3A). Between nine and twelve weeks of gestation (E15–E16 in mice), the epithelial cells of the MEE disappear, leading to mesenchymal confluence of the palate and the completion of palatogenesis. The mechanisms and cellular dynamics of this disappearance have been debated between three primary mechanisms (which may be involved in combination): epithelial-to-mesenchymal transition (EMT), apoptosis/non-apoptotic cell death, and migration to the nasal and oral surfaces [10]. Live imaging studies further support orthogonal cell displacement and epithelial cell extrusion driven by activation of the Rho and myosin light chain kinases, and ultimately the activation of the non-muscle myosin IIA [11]. The completion of palatogenesis by 12 weeks of gestation (E17 in mice) results in the division of the oral cavity into the oral cavity proper and the nasal cavity (Figure 3).

### 2.6. The Periderm

The periderm is a single layer of squamous cells that is considered to be the first stratification event of the epidermis around 55 d EGA. It is distinct from—and precedes—the later-developed process of normal epidermal differentiation, which involves distinct spinous, granular, and cornified layers. In mice, the formation of the periderm begins on the tips of the tail and distal limbs at E9 [12]. Periderm cells are distinct from basal cells from many perspectives: they exhibit an elongated cytoplasmic and nuclear shape, they are unable to retain [^3^H]thymidine for long periods of time (actively cycling), and they express keratin 6, 8, 17, 18, and 19, all characteristics of simple and glandular epithelia [13]. They are thought to originate from the basal layer by delamination as they lose contact with the underlying basement membrane [14]. These cells proceed to cover the facial structures by E10.5 and the intraoral structures soon after [12].

In the context of craniofacial development, the periderm is essential for palatogenesis at two critical developmental stages: First, as the palatine shelves initiate their vertical-to-horizontal transition, this single-cell layer acts as a nonstick barrier, preventing the developing palatine shelves from becoming adherent to the tongue, maxillary epithelium, or mandibular epithelium. Later, once the palatine shelves reorient to the horizontal position and make contact in the midline, the periderm needs to slough off for basal keratinocytes to adhere and fuse [15]. The failure of the periderm to form or function leads to craniofacial defects, discussed later.

## 3. Cellular Adhesions

### 3.1. Overview

Cellular adhesions are the main cellular structures that mediate the integrity of epithelial tissue and confer its barrier function. These cellular structures are present in most tissues, and may be classified according to (1) their ability to interact with the actin cytoskeleton or the intermediate filaments, or (2) whether the adhesions are formed between two neighboring cells or between a cell and the extracellular matrix. Cellular adhesions are highly dynamic and need to be modified and rearranged during morphogenic events such as embryonic development and tissue repair. Mutations impairing the assembly of adhesions or adhesion dynamics result in severe defects, frequently causing early embryonic death.

**Figure 5:**
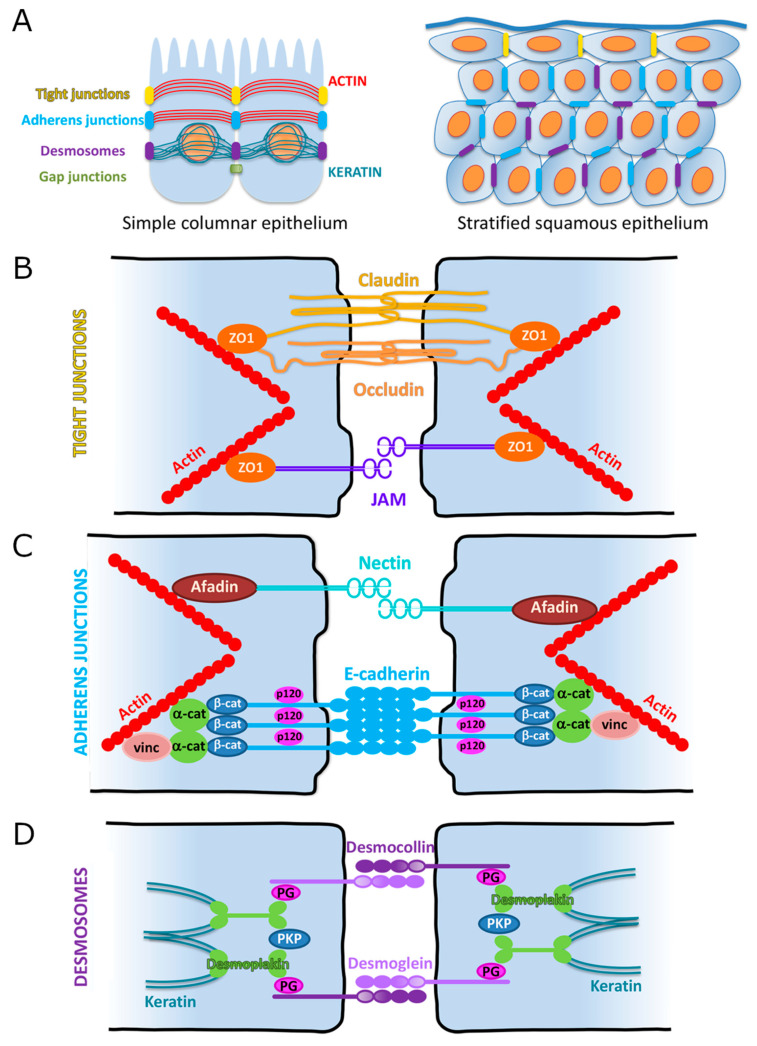
**Schematic representation of cell–cell adhesion junctions in simple columnar and stratified epithelia**. (**A**) Classic organization of cell–cell adhesions between two epithelial cells of a simple columnar epithelium (**left**). Note the apical localization of the tight junctions, superficial to the adherens junctions. Both of these junctions connect to the actin cytoskeleton. Desmosomes are more baso-lateral and connect to the keratin intermediate filaments. This organization is preserved in a stratified epithelium (**right**), with desmosomes and adherens junctions mainly in the basal and suprabasal layers, while the tight junctions are exclusively found in the uppermost layers. (**B**) Schematic of tight junctions. (**C**) Schematic of adherens junctions. α-cat = alpha-catenin; β-cat = beta-catenin; p-120 = p120-catenin; and vinc = vinculin. (**D**) Schematic of desmosomes. PG = plakoglobin; PKP = plakophilin.

This section focuses on cell–cell adhesions. There are four main types of cell–cell adhesions (Figure 5). Tight junctions (TJs, also called *zonula occludens*) and adherens junctions (AJs, also called *zonula adherens*) link the actin cytoskeleton of neighboring cells. Desmosomes (also called *macula adherens*), however, link the intermediate filaments of neighboring cells. Finally, gap junctions are clusters of channels that form tunnels between two neighboring cells.

Where are these adhesion complexes located in epithelial cells? The classic model uses intestinal or renal cells of a simple columnar epithelium as the standard. In this model the TJs are always apico-lateral and the AJs alternate with desmosomes in a more baso-lateral pattern. However, this organization only applies to simple epithelia. In stratified epithelia, such as the lining of the oral cavity or the epidermis, the organization of these junctions has to take into account the presence of multilayered epithelial cells. With this in mind, one can think of the entire stratified epithelium as a simple intestinal cell with TJs stitching upper suprabasal cells together, in addition to AJs and desmosomes connecting basal and lower suprabasal cells (Figure 5A).

### 3.2. Adherens Junctions (AJs)

AJs are one of the best characterized types of cell–cell adhesions. They are comprised of two transmembrane proteins: cadherins and nectins (Figure 5C). Through the extracellular regions of these proteins, they link the actin cytoskeleton of neighboring cells. As such, they confer to cells their ability to sense, generate forces, and become de facto mechanotransducers [16]. Through their intracellular regions they interact with numerous proteins to regulate signaling events, ultimately leading to the regulation of gene expression.

#### 3.2.1. Cadherin-Based Adhesions

Cadherins are transmembrane glycoproteins of 750–900 amino acids that mediate cell–cell adhesion in a homotypic and calcium-dependent manner. The family of classical cadherins includes more than 20 different types, of which epithelial cadherin (E-cadherin) is the most studied, as well as being the focus of this review. As with all classical cadherins its structure comprises extracellular, transmembrane, and intracellular domains [16,17,18,19,20,21]. The large extracellular domain contains five subdomains of approximately 100 residues each. Biochemical studies with Xenopus cadherin indicate that cadherins first form lateral *cis*-dimers with cadherins of the same cell [22]. Upon binding to three ions of calcium, these dimers then form homodimers (*trans*) with cadherins of a neighboring cell [23]. The region responsible for this homophilic recognition is located at the carboxyl terminal end of the first extracellular subdomain [24]. The intracellular N-terminus domain contains approximately 150 residues and is necessary for the adhesion function of E-cadherin [25,26]. The molecules recruited to interact with the cytoplasmic tail are β-catenin and p120-catenin, two molecules belonging to the family of armadillo proteins. The interaction of E-cadherin and β-catenin maintains β-catenin in the cytoplasm, where it activates its non-canonical Wnt-mediated pathway. The interaction of E-cadherin and p120-catenin, however, maintains E-cadherin at the cell membrane by preventing the phosphorylation of key tyrosine residues on the cytoplasmic tail of E-cadherin, which would otherwise send it to recycling endosomes [27,28]. E-cadherin does not directly bind the actin cytoskeleton. Instead, actin filaments bind directly to α-catenin (which can bind to β-catenin) and interact with β-catenin-associated proteins, such as vinculin, to reorganize both the cytoskeleton and the AJ complex (Figure 5C).

E-cadherin is an essential molecule in many morphogenetic processes that take place during embryonic development. It is the first cadherin to be expressed during development, where it participates in the compaction of the eight-cell-stage embryo [29]. Murine embryos lacking the *Cdh1* gene (encoding the E-cadherin protein) do not develop past embryonic day four due to their inability to form trophectodermal epithelium [30]. The transcriptional regulation of E-cadherin is multifactorial, with transcription factors such as RB, c-Myc, and AP-2 promoting its transcription, and Smad interacting protein 1 (SIP1), E12/E47, and members of the Snail family inhibiting its transcription [31,32]. This tight regulation is necessary to control the formation of adhesion complexes [33].

In addition to transcriptional regulation of E-cadherin, its post-translational modifications also affect the function of AJs. Much of the existing data suggest that these modifications occur primarily via the phosphorylation and dephosphorylation of tyrosine residues [34]. The phosphorylation of tyrosine residues results in the loss of cell contacts [35], while the phosphorylation of serine residues by casein kinase II increases the interaction with β-catenin thus maintaining cell contacts [36]. Additional post-translational modifications of β-catenin also affect AJ function: phosphorylation by the activation of Src kinase or the EGF receptor prevents adhesions [37,38,39], whereas its dephosphorylation by leukocyte common antigen-related protein (LAR) or protein tyrosine phosphatase (PTP) increases cell–cell adhesion and prevents cell migration [40,41]. Lastly, cadherin clustering stimulates Rho GTPases [42] to promote AJ dynamics.

#### 3.2.2. Nectin-Based Adhesions

The nectin family contains four members, nectin-1–4 [43,44,45,46], each with cell- and tissue-specific alternative splicing. Nectin-based adhesions, similar to cadherin-based adhesions, are formed by *cis*- and *trans*-interactions with dimers of the same (homo) or different (hetero) member of the family [45,47,48]. Nectins contain immunoglobulin-like extracellular loops that are necessary for their dimerization. Their cytoplasmic C-terminal domain contains a PDZ binding motif that is required to bind afadin, an actin-binding protein that links nectins to the actin cytoskeleton [45,46,48].

Contrary to cadherin-based adhesions, nectins do not require calcium to engage in adhesions [45,48]. As such, nectins are nascent adhesions (likely via the proximity of neighboring cells) that subsequently facilitate the recruitment of proteins required for the assembly of other types of cell–cell adhesions, i.e., cadherin-based adhesions.

### 3.3. Tight Junctions (TJs)

Tight junctions are the most intimate contacts formed between cells (Figure 5A,B). They prevent the diffusion of molecules between adjacent cells and the lateral migration of membrane proteins and lipids. The first protein identified as being associated with TJs was *Zonula Occludens* 1 (ZO-1) [49,50]. Since then, more than 30 proteins have been characterized in association with these junctions [51]. Typically, a tight junction is composed of a transmembrane protein (i.e., claudin, occludin, or a junctional adhesion molecule) and a cytoplasmic protein linked to the actin cytoskeleton (i.e., ZO-1). These proteins interact in the intercellular space with peer proteins from adjacent cells in a homophilic or heterophilic fashion (Figure 5B) [52,53,54].

#### 3.3.1. Claudins

Claudins are the most important component of the tight junctions. They have four hydrophobic transmembrane domains, two extracellular loops, and two cytoplasmic domains that correspond to their amino- and carboxy-terminal ends (Figure 5B). Extracellular loops are critical for homophilic and heterophilic interactions, as well as for the formation of selective ion channels. The C-terminal domain is necessary for the localization of the claudins to the TJs. It contains a PDZ motif through which it binds scaffolding proteins containing PDZ binding domains (i.e., ZO-1) [55,56,57]. Distinct claudins and associated PDZ proteins form different tight junctions, which determine their permeability [58]. In fact, up to 24 claudin isoforms have been identified in humans [59], with claudin-1 and -2 constituting the main components of the TJs [56].

#### 3.3.2. Occludin

Occludin is an enzyme (EC1.6) that contributes to the function of the epithelial barrier. Together with claudins, it is a major component of TJs (Figure 5B). Contrary to claudins, however, occludin is dispensable for the assembly of TJs. Occludin has four transmembrane domains, two extracellular loops, and two cytoplasmic ends. This molecule regulates selective paracellular permeability. The intracellular C-terminus interacts with ZO-1 via the PDZ binding domain and links occludin to the actin cytoskeleton. As a result of alternative splicing several isoforms have been identified, each of which has a unique cellular distribution and interaction with other molecules [60].

#### 3.3.3. Zonula Occludens

Many molecules localize to the intracellular region of tight junctions to modulate and stabilize this type of adhesion. Amongst them, Zonula Occludens (ZO) 1, ZO2, and ZO3 are the main components of this intracellular network. These three proteins co-immunoprecipitate with each other and share a similar structure and functional domains. However, they are distinct due to their unique C-terminal regions, which define their specific functions [33,61,62]. ZO1 is usually considered the central molecule responsible for scaffolding and organizing the cytoplasmic events associated with TJs. ZO1 is a 220 kDa protein containing multiple functional domains, among which are PSD95, DlgA, ZO1 homology (PDZ) domains, SRC homology 3 (SH3) domains, and a guanylate kinase homology (GUK) domain. These domains define the specific functions of ZO1. For example, the PDZ, GUK, and C-terminal domains mediate the interaction with claudin, JAMs, occluding, and F-actin, respectively [63,64]. Alternative splicing confers tissue specificity of these components [61].

#### 3.3.4. Junctional Adhesion Molecules (JAMs)

Junctional adhesion molecules (JAMs) are proteins that belong to the superfamily of immunoglobulins. They are subdivided into four types: JAM-A, JAM-B, JAM-C, and JAM-L/JAM4 [65]. From a structural perspective, JAMs have an extracellular domain, a single transmembrane domain, and a cytoplasmic domain. The extracellular domain plays an important regulatory role by being able to bind multiple ligands, which have been proposed to regulate cellular functions and paracellular permeability [66]. The C-terminal region contains a PDZ binding domain (except for JAM-L/JAM4). This domain serves as an anchor to the actin cytoskeleton and to many TJ-associated scaffolding molecules, including ZO1 and the polarity protein Par3. These interactions are necessary for the proper function of the TJs [67,68]. JAM-A is necessary for the formation and assembly of TJs in epithelial cells in a homophilic pattern [69]; however, other studies have shown heterophilic interactions among the JAM family members as well as with other components of adhesion complexes [70,71].

### 3.4. Desmosomes

In 1920 Josef Schaffer coined the term “Desmosome”. It has its origins in the Greek words for bond (*desmo*) and body (*soma*). Also called *macula adherens* (Latin for “adherent spot”), a desmosome is a “binding body”, or a cell structure specialized for cell-to-cell adhesion randomly arranged along the plasma membrane. Desmosomes are one of the strongest cell adhesion types and are found in tissue that experience high mechanical stress, such as epithelia and cardiac muscle. Desmosomes share some structural organization with AJs and TJs: a transmembrane region formed by desmosomal cadherins (desmoglein and desmocollin) that interact in the intercellular space with neighboring cells, and a series of cytoplasmic adaptor proteins that link the cadherins to the cytoskeleton. Desmosomes are also quite distinct from other adhesion complexes. First, desmosomes link the cytoskeletal intermediate filaments (i.e., keratins in epithelia) of adjacent cells, as opposed to the actin filaments. Second, their cytoplasmic proteins are organized in two plaques: the outer dense plaque, where the N-terminus domain of desmoplakin binds the desmosomal cadherins, and the inner dense plaque, where the C-terminus domain of desmoplakin binds the intermediate filaments (inner dense; Figure 5D) [72,73,74]. The presence of desmoplakin as an intermediate between the cadherins and the cytoskeleton is likely to be responsible for the increased strength of the desmosomal adhesion.

The molecular composition of desmosomes is variable, showing tissue- and cell-specific isoforms, conferring functional specificity based on the physiological context [75]. As with AJs, extracellular calcium promotes the assembly of desmosomal proteins [76,77], a characteristic that has been extensively used in keratinocyte cultures to study their dynamics [78].

### 3.5. Desmosomal Cadherins (Desmogleins, Desmocollins)

Desmosomal cadherins belong to the superfamily of cadherins. They contain five extracellular domains and have calcium binding motifs. In the presence of calcium the extracellular domains become rigid and promote adhesion [79,80]. The cytoplasmic tail of these cadherins bind to plakoglobin and plakophilin, members of the armadillo protein family [81]. To date, four desmoglein isoforms and three desmocollin isoforms have been reported. In stratified epithelia, some of these isoforms are ubiquitously detected throughout the tissue (desmoglein 1, desmocollin 2), whereas others show a more restricted localization to specific cell layers (desmoglein 2–4, desmocollin 1, 3, and 4) [82,83]

### 3.6. Desmosomal Armadillo Proteins (Plakophilin, Plakoglobin)

Armadillo proteins are characterized by a central domain containing α-helix-forming repeated units of ~42 amino acids, initially characterized in the Drosophila segment polarity protein armadillo [84,85,86]. Armadillo repeat units fold together as a superhelix, thus creating a functionally rich region able to interact with many protein partners that contribute to the diverse range of functions associated with this family of proteins. In the desmosomes, plakophilin-1–3 and plakoglobin (also named γ-catenin) anchor the cytoplasmic tail of desmosomal cadherins to desmoplakin in the outer dense plaque. In contrast to cadherins, their tissue distribution is uniform throughout stratified epithelia.

### 3.7. Desmoplakin

Desmoplakin is the most abundant and essential component of desmosomes [87]. It belongs to the family of plakins and binds to intermediate filaments in the inner dense plaque [88,89]. Its N-terminal globular head domain is required for both the localization to the desmosome and the interaction with armadillo proteins and desmosomal cadherins [88]. The C-terminal region is composed of three plakin repeats essential for binding to the intermediate filaments [88]. Serine phosphorylation of the C-terminal region inhibits interaction with the intermediate filaments, ultimately disassembling the adhesion complex [90,91].

## 4. Contribution of Cellular Adhesion to Craniofacial Morphogenesis

**Figure 6:**
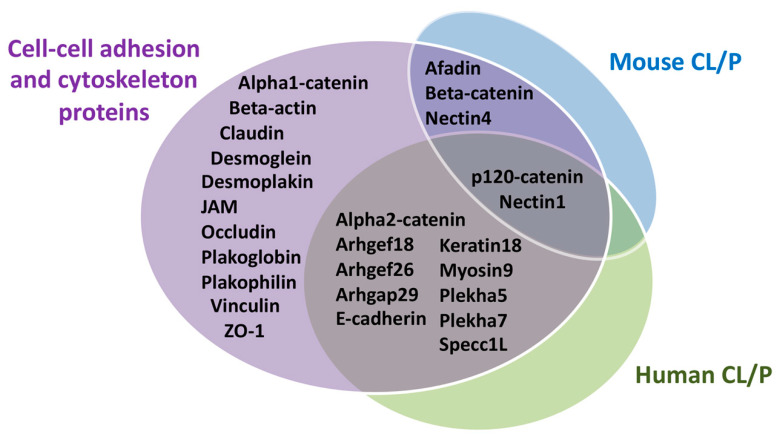
**Summary of cell–cell adhesion and the cytoskeletal proteins involved in human and mouse cleft lip with or without palate**. Adhesion molecules discussed in this review were distributed on the basis of whether they played a role in human cleft lip with or without palate (CL/P), mouse CL/P, both, or had no identified role in craniofacial morphogenesis.

Because of their critical role in the assembly of individual cells into three-dimensional tissues, cellular adhesions contribute to every step of craniofacial morphogenesis. This includes the formation and delamination of neural crest cells and their migration into pharyngeal arches, the formation and expansion of facial processes, and the fusion of these processes to form the face. We will limit our review to the later stages of facial morphogenesis with an emphasis on the formation of the lip and the palate. We will first review the contribution of the cytoskeleton and then take a more systemic approach by evaluating the role of adhesion molecules in facial morphogenesis. We will learn from human genetic studies of individuals with cleft lip with or without cleft palate (CL/P) and then from animal models to highlight the contribution of cellular adhesion to facial morphogenesis. A summary of these intersecting genes is presented in Figure 6 and Table 2.

### 4.1. Cytoskeleton in Craniofacial Morphogenesis

The remodeling of the cytoskeleton is essential during morphogenesis. During the formation of the lip and the palate, two main cell types undergo dynamic remodeling: ectodermal-derived epithelial cells and neural-crest-derived mesenchymal cells. Amongst the filaments constituting their cytoskeleton, both populations contain actin and microtubules. However, their intermediate filaments are distinct, with epithelial cells characterized by keratin filaments while mesenchymal cells contain vimentin. Although genetic variants in the *ACTIN* gene are not associated with orofacial clefting, early studies demonstrated the active production of actin in palatal shelves as they transition from a vertical to a horizontal position [92], supporting the dynamic remodeling of the cytoskeleton. During that process mesenchymal cells align their actin cytoskeleton, further demonstrating an actin-dependent cell contractility that drives palatal shelf elevation [93]. Actomyosin contractility is also required during the fusion of the secondary palatal shelves, as cells from the medial epithelial seam intercalate to form a single-layered epithelium concomitantly with epithelial cell extrusion [11]. Not surprisingly, genetic variants in the *MYOSIN9* gene have been reported in the study of families with orofacial clefts and found to be associated with an increased risk for the defect [94,95,96]. Novel variants in the *KRT18* gene have also been shown to contribute to non-syndromic CL/P (NSCL/P) [97]. Although keratins can remodel the cytoskeleton via desmosomal adhesions, the association of *KRT18* to NSCL/P may be more related to its putative function in the periderm [98] than to a role in remodeling the cytoskeleton.

**Table 2:** Summary comparison of cell adhesion molecules, cleft lip with or without palate in human, and murine knockout phenotypes.

Gene (Protein)	Type of Human Craniofacial Clefts	Human Variants Associated with the CL/P Phenotype	Mouse Knockouts Craniofacial Phenotype
AFDN (Afadin)	No CL/P		KO embryonic lethal [99] K14-Cre cKO no CP [100] In utero cKO CP and oral adhesions [100]
CTNNA2 (Alpha2-catenin)	NSCL/P [101]	g.82025185	None
ARHGEF18 (Arhgef18)	NSCL/P [102]	c.1484G>A p.Arg495Gln	None
ARHGEF26 (Arhgef26)	NSCL/P [103]	g.153840512:A>T g.153943770:C>G	None
ARHGAP29 (Arhgap29)	NSCL/P [104,105,106,107]	c.62_63delCT p.Ser21Tyrfs*20 c.91C>T p.Leu31Phe c.698-1G>C c.888G>C p.Arg296Ser c.976A>T p.Lys326* c.1252G>A p.Val418Ile c.1475C>A p.Ser492* c.1576+1G>A c.1654T>C p.Ser552Pro c.1847G>A p.Arg616His c.1865C>T p.Thr622Met c.2017T>G p.Phe673Val c.2109+1G>A c.2393G>A p.Arg798Gln c.2533A>G p.Ile845Val c.2617C>T p.Arg873Cys c.3023G>A. p.(Arg1008Lys) c.3326_3328delCAA p.Thr1109del c.3339T>G p.Ile1113Met g.94545160:T>C g.94547883:C>G g.94547889:G>A	KO embryonic lethal [108] Heterozygote no CP but oral adhesion [108]
CTNNB1 (Beta-catenin)	No CL/P [109]		cKO CP [110]
CTNND1 (p120-catenin)	Syndromic CL/P [111] NSCL/P [112,113]	c.606_627del p.Pro203Leufs*25 c.1093C>T p.Gln365* c.2098C>T p.Arg700* g.57559005:C>G p.Gln19Glu g.57564445_5756446del p.Asp313Profs*9 g.57569255:G>A p.Trp336* g.57571168:A>G p.Asp499Gly g.57572202:C>T p.Leu558Phe g.57573381:C>T p.Arg584Trp g57575761:G>T p.Try690Cys g.57576939:G>T c.2417+1G>T g.57578892:C>T p.Arg852* c.1381C>T p.Arg461* c1481_1485del p.Leu494Argfs*5 c.2598_2601dupTGAT p.Ser868* c.2737dupC p.His913Profs*3	CreCT cKO 47% CP [112]
CDH1 (E-cadherin)	Syndromic CL/P [111] NSCL/P [112,114,115,116,117,118]	c.760G>T p.Asp254Tyr c.770A>T p.Asp257Val c.1320G>T p. ? c.1320+1G>C p. ? c.1361_1363del p.Val454del c.387+5G>A p.? c.468G>C p.Trp156Cys c.752C>T p.Thr251Met c.760G>A p.Asp254Asn c.768T>A p.Asn256Lys c.1023T>G p.Tyr341* c.1489G>A p.Glu497Lys c.1766A>T p.Asn589Ile c.2351G>A p.Arg784His c.2426_2427del p.Asn809Ilefs*3	KO embryonic lethal [119] Wnt1-Cre cKO no cleft [120] K14-Cre cKO no cleft [121]
KRT18 (Keratin18)	NSCL/P [97]	g.53344318:G>T	None
MYOSIN9 (Myosin9)	NSCL/P [94,95,96]	g.35044605:C>T g.35048804:C>T g.35007860:T>C	None
PVRL1 (Nectin1)	Syndromic CL/P [122]	p.Tryp185X	KO no CL/P [123,124] In utero cKO CP [100]
PVRL4 (Nectin4)	No CL/P [125]		KO 11–40% CP [100]
PLEKHA5 (Plekha5)	NSCL/P [112]	g.19440414:A>G p.Tyr590Cys	None
PLEKHA7 (Plekha7)	NSCL/P [112]	g.16838582:C>T p.Gly544Asp g.16838676:G>A p.Arg513Trp g.16834682:T>C p.Asp662Gly	None
SPECC1L (Specc1L)	Syndromic CL/P [126] NSCL/P [127]	c.569C>T p.Thr190Met c.1244A>C p.Gln415Pro c.273G>A p.Met91Iso c.256G>A p.Ala86Thr c.895A>G p.Thr299Ala c.1637G>A p.Arg546Gln	KO embryonic lethal [127] In-frame deletion CP [128]

### 4.2. Role of Adherens Junctions in Facial Morphogenesis

#### 4.2.1. Cadherin-Based Adhesions: Lessons from Patients

One of the most important components of AJs is E-cadherin, encoded by the *CDH1* gene. Missense mutations and rare variants in *CDH1* were identified in patients with non-syndromic CL/P [112,114,115,116,117], patients with hereditary diffuse gastric cancer and CL/P [118], as well as in one of the syndromic forms of CL/P (blepharocheilodontic syndrome, BCDS [111]). Interestingly, most pathogenic CL/P variants cluster in the linker regions between the extracellular domains of E-cadherin [118], likely affecting its chelation to calcium and altering its function.

In the cytoplasm, E-cadherin interacts with beta-catenin (encoded by the *CTNNB1* gene), which is also a major player in the Wnt signaling pathway. De novo nonsense and frameshift mutations in *CTNNB1* were reported in patients with craniofacial phenotypes, but they did not present with CL/P [109], suggesting that in humans mutations in *CTNNB1* are not directly associated with CL/P. However, many other members of the Wnt signaling pathway have been associated with CL/P, including numerous Wnt ligands, Wnt receptors (Frizzled), and co-receptor ROR2 (for a full review of the Wnt signaling contribution to orofacial clefting, see [129]).

Two other catenins are part of AJs: p120-catenin (encoded by the *CTNND1* gene), which anchors E-cadherin at the plasma membrane, and α-catenin (encoded by the *CTNNA1* gene), which mediates forces and interacts with the actin cytoskeleton. Genetic variants in *CTNND1* cause blepharocheilodontic syndrome [111] and increase the risk for non-syndromic CL/P [112,113]. Novel protein-truncating variants and de novo variants were also identified in families with craniofacial dysmorphisms and cardiac, limb, and neurodevelopmental anomalies [113]. Some of these variants affected the binding of p120-catenin to E-cadherin [112,113], suggesting an alteration in the AJ complex and cellular adhesions in these individuals. To the best of our knowledge, no genetic variants in *CTNNA1* have so far been reported in association with CL/P. However, variants in *CTNNA2* were identified in consanguineous families with NSCL/P [101]. This isoform is typically detected in the brain, reinforcing previous evidence of a correlation between neuronal and craniofacial defects [130,131].

#### 4.2.2. Cadherin-Based Adhesions: Lessons from Murine Models

Because of its prominent role in cellular adhesion, animals deficient in E-cadherin do not undergo morphogenesis and are embryonic lethal [119]. However, animals with a conditional knockout of E-cadherin in neural crest cells survived embryonic development and exhibited craniofacial defects related to skeletal development, but no cleft palate [120]. Similarly, cleft palate was not observed when E-cadherin was ablated in epithelial cells using a keratin 14-cre driver [121]. Conditional ablation of p120-catenin using an ectoderm-specific Cre driver (CreCT) resulted in 47% of animals with overt clefts, while the remaining animals exhibited delays in medial growth of the maxillary labial processes [112]. Conditional knockout and conditional gain of function of β-catenin in palatal epithelial cells, however, led to cleft palate [110]. The higher penetrance of a cleft palate phenotype in animals with altered levels of β-catenin may be due not to its role as a component of the AJ complex (non-canonical) but as a transcription factor (canonical) with many downstream effectors, a property not associated with p120-catenin or E-cadherin.

#### 4.2.3. Nectin-Based Adhesions: Lessons from Patients

Although nectin-based adhesions have not been extensively studied compared to AJs or TJs, mutations in both *NECTIN1* (encoded by the *PVRL1* gene) and *NECTIN4* (encoded by the *PVRL4* gene) cause syndromes involving orofacial clefts (*NECTIN1*) or phenotypes previously associated with clefting (*NECTIN4*), supporting their critical role in facial morphogenesis. *PVRL1* was identified as the gene responsible for the autosomal recessive CLP-ectodermal dysplasia (ED) syndrome (CLPED1, previously ED4), characterized by CL/P, hidrotic ED, developmental defects in the hands, and occasional mental retardation [122]. Homozygous nonsense (W185X) and frameshift mutations identified in patients truncate the nectin-1 protein and abolish its interaction with afadin, likely abolishing the calcium-independent cell–cell adhesion complex [122]. Mutations in *PVRL4* cause ED syndactyly syndrome 1 (EDSS1, previously ED1), a syndrome similar to CLPED1 yet distinct because of a lack of orofacial defects [125]. These mutations affect different protein interaction domains, including the afadin binding site [125]. To date, no human genetic variant in the *AFADIN* gene has been reported to be associated with orofacial clefting; however, only 25% of clefting heritability is currently known [132].

#### 4.2.4. Nectin-Based Adhesions: Lessons from Murine Models

Despite the clear evidence that mutations in *PVRL1* cause a syndromic form of CL/P in humans, murine models have failed to recapitulate this phenotype. In fact, no CL/P was observed in mice homozygous null for *Nectin-1* or in compound mutants where *Nectin-1* and -*3* were deleted [123,124]. Recently, Lough et al. revisited the loss of nectin-1 and nectin-4 in mice. Rather than deleting these alleles using homologous recombination or tissue-specific Cre drivers, the investigators took advantage of an in utero lentiviral-mediated genetic approach to knockdown these proteins [100]. The loss of *Nectin-1* and the loss of *Nectin-4* led to cleft palate with a penetrance of 11–40%. Interestingly, synergistic dual loss of *Nectin-1* and *Nectin-4* caused highly penetrant cleft palate, with oral adhesions between the palatal shelves and the tongue due to the partial loss of periderm cells [100]. Because afadin is an obligate binding partner of nectin function [133], one would expect orofacial clefting in an afadin loss-of-function model. Germline homozygous null mice were early embryonic lethal [99], preventing the evaluation of afadin’s role in facial morphogenesis, while the epithelial-specific loss of afadin (using a K14-Cre driver) showed proper palatogenesis [100]. However, lentiviral-mediated delivery of the Cre recombinase a few days earlier than the onset of the K14-Cre driver led to the failure of palatal shelf elevation and fusion due to intraoral adhesions [100]. Collectively, these studies are important for two reasons: First, they highlight the importance of the timing of the delivery of the Cre recombinase in tissue-specific knockout and warrant the potential re-examination of mutants where the phenotype of the murine model did not phenocopy the human phenotype. Second, they reinforce the role of the periderm in palatogenesis. Amongst the different nectins, nectin-4 is specific to the periderm. Its loss, or the loss of its binding partner, afadin, showed strong intraoral adhesions, further supporting the theory of a role for nectin-based adhesions in facial morphogenesis.

### 4.3. Role of Other Adhesion Components in Facial Morphogenesis

#### 4.3.1. Lessons from Patients

Despite the fact that TJs anchor to the actin cytoskeleton and that actin cytoskeleton remodeling is necessary for facial morphogenesis, to the best of our knowledge no human genetic variants in any of the proteins comprising the TJ complex have been associated with CL/P. Similarly, none of the desmosomal proteins, critical for remodeling the keratin of epithelial cells, have been associated with CL/P. The reason for this lack of association in not clear, but one could speculate that the role of TJs in barrier formation is so critical that a small defect would be incompatible with life. As for a lack of desmosomal contribution to facial morphogenesis, we may want to consider an evolutionary perspective. Desmosomes are sophisticated structures that appeared with the appearance of stratified epithelia [83]. Palatogenesis occurs at a time when the oral epithelium resembles a simple epithelial sheet, in which the main form of cell-to-cell junctions is AJs. The appearance of desmosomes would occur after the palate has formed, potentially diminishing the role of desmosomes in this morphogenic event.

#### 4.3.2. Lessons from Murine Models

We investigated the Mouse Genome Informatics database for “craniofacial phenotype”, “abnormal craniofacial development”, and “abnormal palate development”. From the 192 genotypes corresponding to these phenotypes, none were deficient in desmosomal or tight junction proteins.

### 4.4. Other Molecules Modulating Cell–Cell Junctions Critical for Facial Morphogenesis

#### 4.4.1. SPECC1L and Pleckstrin

Amongst the numerous proteins that interact with actin and microtubules, three showed an association with human CL/P. The first is *SPECC1L* (sperm antigen with calponin homology and coiled-coil domains 1 like). Mutations in this gene are responsible for oblique facial clefts [126], a rare form of orofacial clefts [134], and are clustered in the second coiled-coil and calponin homology domains. Human genetic variants outside of the coiled-coil domain were also reported in individuals with non-syndromic CL/P and resulted in milder functional defects than those of variants associated with syndromic clefts [127]. The loss of *SPECC1L* in mice resulted in early embryonic lethality. However, mice homozygous for a truncation allele showed an altered palatal rugae phenotype with oral adhesions and a reduction in interferon regulatory factor 6 (IRF6) in these structures, but no cleft palate [127]. Homozygotes for in-frame deletions in the second coiled-coil domain resulted in exencephaly, cleft palate, and ventral body wall closure defects [128], demonstrating the essential functional role of the coiled-coil domain. In vitro, functional studies demonstrated that SPECC1L colocalized with microtubules and filamentous actin in palatal mesenchymal cells [126]. Deletions or mutations in the coiled-coil domain severely affected its ability to associate with microtubules, reducing its ability to traffic in the cells and resulting in a perinuclear accumulation. This uneven distribution of SPECC1L in the cells resulted in increased actin and non-muscle myosin II bundles at the cell periphery [128]. Ultimately, the disrupted actomyosin cytoskeleton would affect cell alignment and coordinated movement required for elevation and fusion of the palatal shelves.

The other two cleft- and microtubule-associated proteins are pleckstrin-homology-domain-containing protein 5 and 7 (PLEKHA5 and PLEKHA7). These molecules are cytoplasmic accessory members of AJs. Although very little is known about the function of PLEKHA5, studies show that PLEKHA7 contributes to the integrity of AJs by linking the E-cadherin/p120 catenin complex to the minus end of microtubules [135]. It also stabilizes nectin-based junctions by recruiting the PDZD11 protein to the adhesion complex. Although mice deficient in *Plekha7* exhibited normal gross morphology, including craniofacial appearance, genetic variants in both *PLEKHA5* and *PLEKHA7* were associated with CL/P in multiple populations [112]. Interestingly, genetic variants in *PLEKHG3*, another family member related to PLEKHA5 and PLEKHA7, were also reported in association with CL/P [103], further supporting the theory of a role for these cytoplasmic proteins in the cell–cell adhesion stability required for proper facial morphogenesis.

#### 4.4.2. IRF6 and the Rho Pathway

In addition to molecules that directly participate in the formation of an adhesion complex, some transcription factors and growth factors are essential modulators of cellular adhesion and are involved in syndromic and non-syndromic orofacial clefts. One such transcription factor is interferon regulatory factor 6 (IRF6). Mutations in *IRF6* cause Van der Woude syndrome, the most common syndromic form of CL/P, in which the vast majority of patients present with a cleft lip with or without cleft palate and lip pits in their lower lips [136]. Genetic variants in *IRF6* also contribute to an increased risk for non-syndromic CL/P [137], making IRF6 one of the prominent contributors to CL/P. No patients have been reported with homozygous *IRF6* mutations, likely because of the fact that the loss of IRF6 would be incompatible with life, as demonstrated by animal models. Indeed, *IRF6*-deficient mice die postnatally from dehydration and an inability to suckle, due to the absence of an epidermal barrier and a fused oral cavity [138]. Further investigation into the cause of oral fusion demonstrated that *IRF6*-deficient embryos lack an oral periderm, allowing abnormal tissue adhesions between the tongue and palatal shelves or between two opposing maxillary and mandibular oral epithelia [12,138]. In the absence of IRF6, E-cadherin, which is normally restricted to the lateral and basal sides of the most superficial layers of oral epithelial cells, was detected on the apical side. This ectopic localization was due in part to altered tight junctions, and promoted the formation of adhesion complexes with opposing epithelial cells [12]. In vitro, our recent studies indicate that IRF6 regulates the delivery of E-cadherin to the plasma membrane [139]. How IRF6 participates in E-cadherin turnover is not fully elucidated, although its association with NME1/2 could contribute to adhesion protein trafficking or recycling to the plasma membrane [112]. Other adhesion molecules were reduced at the plasma membrane of *IRF6*-deficient keratinocytes, supporting a more global effect of IRF6 on cellular adhesions.

In addition to its role in tissue and cellular adhesions, IRF6 inhibits the small GTPase RhoA [140], a major signaling node that influences cytoskeleton changes downstream of cell adhesions [141,142]. RhoA cycles between an active-GTP bound (activated by guanine nucleotide exchange factors, GEFs) and an inactive-GDP bound form (inactivated by RhoA GTPase-activating proteins, GAPs). In the absence of IRF6, keratinocytes exhibited increased stress fibers and reduced migration [140]. During this process we found that one of the GAPs, namely ARHGAP29, was downregulated [140]. This is significant because human genetic variants in *ARHGAP29* have been associated with CL/P in numerous populations [104,105,106,107]. In addition, although *ARHGAP29* homozygous null embryos die early during embryogenesis before facial morphogenesis, mice heterozygous for a patient-derived mutation knock-in allele presented with abnormal oral epithelial adhesions [108]. ARHGAP29 also plays a role in the migration of endothelial cells by binding afadin to regulate Rho-associated kinase activity [143]. Collectively, these studies highlight a critical role for IRF6 in regulating the actin cytoskeleton by modulating RhoA activity during craniofacial morphogenesis. Other GAPs and GEFs, in addition to ARHGAP29, have been associated with CL/P in humans. Variants reported in *ARHGEF18* [102] and *ARHGEF26* [103] reinforce the fundamental role of actin cytoskeletal remodeling during craniofacial development.

## 5. Conclusions

Cells are the base units of all organisms. They touch, adhere, and engage their cytoskeletons in response to each other’s shape. Cells also move, both alone and collectively, following cues to ultimately form structures. These cues vary in their nature, ranging from a leader cell who is secreting growth factors to the stiffness of extracellular matrices. They also include forces exerted by neighboring cells and sensed by target cells via adhesion receptors. By reviewing the evidence linking gene regulatory networks involved in cellular adhesions with orofacial morphogenesis, it becomes clear that a more integrated approach that includes biomechanical processes—mediated in part by cellular contacts—will be essential to further our understanding of in vivo embryonic development. This will require the generation of new tools to measure forces in vivo as embryos develop in addition to improved live imaging to track cells as they move within increasingly complex structures.

## Figures and Tables

**Table 1:** Derivatives of pharyngeal arches.

**Pharyngeal Arch**	**Ectoderm**	**Endoderm**	**Mesoderm**				**Neuroectoderm**	
	*Pharyngeal cleft*	*Pharyngeal pouch*	*Skeletal*	*Visceral*	*Arterial*	*Muscular*	*Motor* *nervous*	*Sensory* *nervous*
**First (mandibular)**	External acoustic meatus, helical crus, tragus (anterior 3 hillocks of His)	Auditory tube, tympanic membrane *	Mandible (Meckel’s cartilage), maxilla ^ƒ^, palatine bone ^ƒ^, malleus, incus, teeth	Body of tongue (anterior 2/3)	External carotid, maxillary	Muscles of mastication, tensor tympani, tensor veli palatini ^, mylohyoid, anterior belly of digastric	CN V, maxillary division V3	CN V, lingual nerve
**Second (hyoid)**	Helix, antihelix, antitragus, lobule (posterior 3 hillocks of His)	Tonsillar fossa	Stapes, styloid process, superior hyoid body	Midtongue, thyroid, tonsil	Stapedial	Muscles of facial expression, stapedius, hyoid, posterior belly of digastric	CN VII	CN VII, chorda tympani (taste)
**Third**	----	Inferior parathyroid, thymus	Inferior hyoid body, great cornu hyoid	Root of tongue (posterior 1/3), epiglottis, thymus, carotid body	Internal carotid	Stylopharyngeus	CN IX	CN IX
**Fourth**	----	Superior parathyroid	Thyroid and laryngeal cartilages	Epiglottis, superior parathyroid	Aorta (left), subclavius (right)	Pharyngeal constrictors, levator veli palatini ^, palatoglossus ^, palatopharyngeus ^	CN X, superior laryngeal	Auricular nerve to external acoustic meatus
**Fifth**	----	----	----	----	----	----	----	----
**Sixth**	----	Telopharyngeal body, parafollicular (“C”) cells	Cricoid, aretynoid, corniculate cartilages	Larynx	Pulmonary arteries, ductus arteriosus	Cricothyroid, laryngeal muscles, pharyngeal constrictors	CN X, inferior laryngeal	CN X

* Note that the pharyngeal pouch of the first pharyngeal arch is derived from the ectoderm, as illustrated in Figure 1C′. ^ƒ^: component of the hard palate. ^: component of the soft palate.

## Data Availability

Not applicable.
